# A simple-to-use web-based calculator for survival prediction in Parkinson’s disease

**DOI:** 10.18632/aging.202443

**Published:** 2021-02-01

**Authors:** Yunliang Tang, Jiao Wang, Gengfa Chen, Wen Ye, Nao Yan, Zhen Feng

**Affiliations:** 1 Department of Rehabilitation Medicine, First Affiliated Hospital of Nanchang University, Nanchang 330006, Jiangxi, China; 2 Department of Endocrinology and Metabolism, First Affiliated Hospital of Nanchang University, Nanchang 330006, Jiangxi, China; 3 Department of Neurology, Zhongnan Hospital of Wuhan University, Wuhan 430071, Hubei, China

**Keywords:** Parkinson’s disease, nomogram, calculator, survival, mortality

## Abstract

Background: To establish and validate a nomogram and corresponding web-based calculator to predict the survival of patients with Parkinson’s disease (PD).

Methods: In this cohort study, we retrospectively evaluated patients (n=497) with PD using a two-stage design, from March 2004 to November 2007 and from July 2005 to July 2015. Predictive variables included in the model were identified by univariate and multiple Cox proportional hazard analyses in the training set.

Results: Independent prognostic factors including age, PD duration, and Hoehn and Yahr stage were determined and included in the model. The model showed good discrimination power with the area under the curve (AUC) values generated to predict 4-, 6-, and 8-year survival in the training set being 0.716, 0.783, and 0.814, respectively. In the validation set, the AUCs of 4- and 6-year survival predictions were 0.85 and 0.924, respectively. Calibration plots and decision curve analysis showed good model performance both in the training and validation sets. For convenient application, we established a web-based calculator (https://tangyl.shinyapps.io/PDprognosis/).

Conclusions: We developed a satisfactory, simple-to-use nomogram and corresponding web-based calculator based on three relevant factors to predict prognosis and survival of patients with PD. This model can aid personalized treatment and clinical decision-making.

## INTRODUCTION

Parkinson’s disease (PD) is a progressive neurodegenerative disorder mostly defined by motor symptoms such as resting tremor, muscular rigidity, bradykinesia, and postural instability [1]. Globally, an estimated 6 million people suffer from PD, and this number is expected to double by 2040, making PD the world’s fastest-growing neurological disorder [2]. The majority of PD cases are idiopathic, although 10-15% are genetic, known as familial PD [3]. Although its precise etiology remains unclear, the pathophysiological basis is degeneration of dopaminergic neurons in the substantia nigra [4]. Current treatment for PD involves pharmaceutical, surgical, and dietary interventions, and/or rehabilitation exercises [5]. Dopamine replacement strategies have been the most effective pharmacotherapy for motor symptoms in PD [6]. Subthalamic nucleus deep brain stimulation is a particularly promising new therapy, shown to effectively improve not only motor function but also executive function in PD [7]. However, there is no well-established treatment to stop or slow the disease progression in PD patients. Rapid PD progression is associated with a poor prognosis [8]. Therefore, there is an urgent need for reliable and accurate prognostic models for PD.

Numerous studies have aimed to discover potential PD biomarkers, including physiological or biochemical measurements, metabolic and genetic assessments, imaging findings, and rating scales that could represent candidate indicators [9–11]. For instance, Majbour et al. reported that cerebrospinal fluid alpha-synuclein species correlated with the progression of motor impairment [12], Chung et al. constructed a model to predict later development of gait freezing in PD [13], and Hideyuki et al. reported that high C-reactive protein (CRP) levels were associated with shorter survival time and suggested that they may predict survival prognosis in patients with PD [14]. Although considerable effort has been made, few of these biomarkers have been adopted in routine clinical practice. More importantly, to the best of our knowledge, there is no effective prognosis prediction model for patients with PD.

In the present study, we aimed to establish a model by using Cox regression based on long-term follow-up of the cohort to predict the prognosis of PD patients. We first established a nomogram based on the primary predictive model and it was displayed as a web-based calculator for convenient clinical use. The predictive value of the model was evaluated based on discrimination, calibration, and clinical utility in the training and validation sets. This simple-to-use model might serve as an early warning and prediction system for patients with PD.

## RESULTS

### Baseline patient characteristics

**Figure 1:**
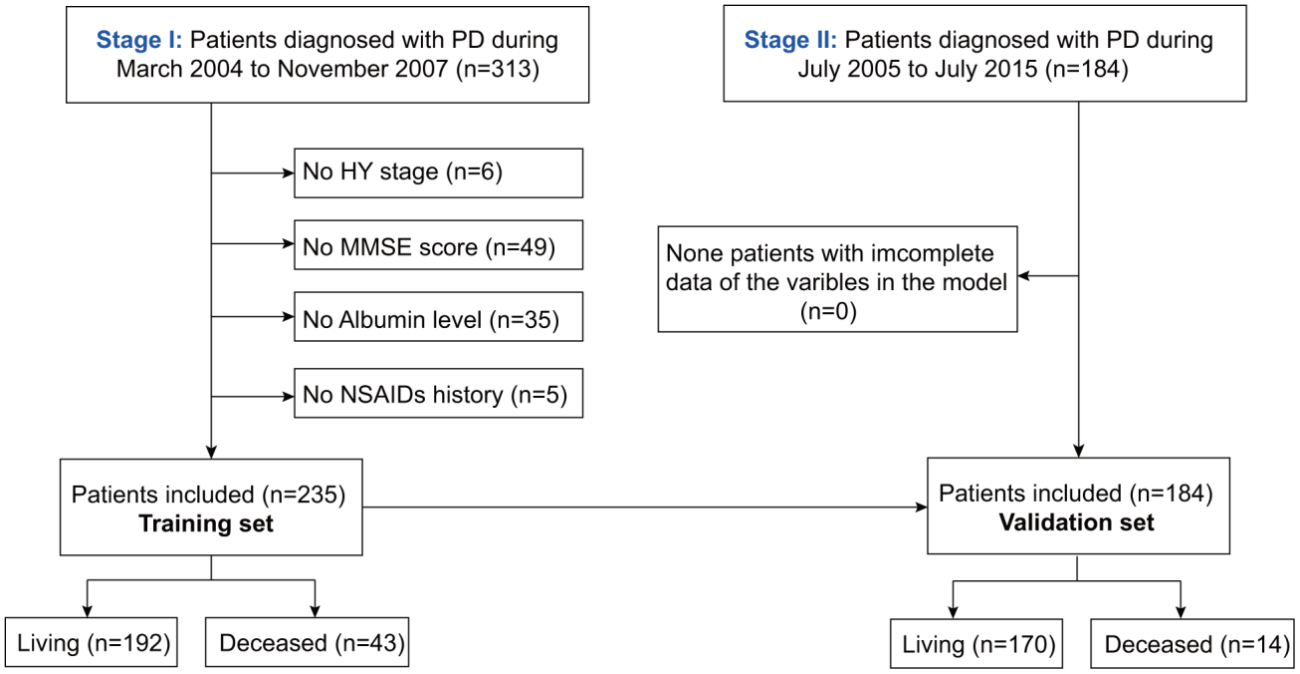
**Flowchart of participant selection.**

As shown in Figure 1, after excluding patients with unknown HY stage, MMSE scores, albumin, or NSAIDs history, the rest were included in the training (n=235) and validation sets (n=184). The baseline characteristics of the training and validation sets are described in Table 1. The median follow-up times in the training and validation sets were 69.3 (2.9, 118.9) and 19.3 (1.8, 79.3) months, respectively. During the follow-up period, 43 patients died and 192 survived in the training set. Further, the mortality in the training set after 4, 6, and 8 years was 24.4%, 26.8%, and 20.7%, respectively. In the validation set, 17 patients died and 170 survived. Additionally, the mortality in the validation set after 4 and 6 years was 7.1%, and 7.7%, respectively.

**Table 1:** Baseline characteristics of the training set and validation set.

**Features**	**Training set (n=235)**	**Validation set (n=184)**	**P-value**
Age (years)	69.25±9.12	72.97±8.06	<0.001
Sex			0.006
Male	100 (42.6%)	104(56.5%)	
Female	135 (57.4%)	80(43.5%)	
PD duration (years)	7.90±5.44	8.59±5.63	0.204
HY			0.547
≤3	148 (63.0%)	122 (66.3%)	
≥4	87 (37.0%)	62 (33.7%)	
MMSE	24.42±4.98	24.38±5.83	0.947
Albumin (mg/dL)	4.03±0.41	3.68±0.45	<0.001
CRP			-
<0.3mg/L	70 (29.8%)	-	
0.3-0.8mg/L	93 (39.6%)	-	
>0.8mg/L	72 (30.6%)	-	
NSAIDs			-
Non	195(83.0%)	-	
Current/habitual	40(17.0%)	-	
Vital status			0.002
Alive	192(81.7%)	170(92.4%)	
Deceased	43 (18.3%)	14 (7.6%)	

### Prognostic factors in PD

Four variables including age, PD duration, Hoehn-Yahr (HY), and albumin, were significantly associated with overall survival as per the univariate regression analysis. Thereafter, three variables (age, PD duration, and HY) were identified as independent prognostic factors for PD by multivariate Cox regression analysis (Table 2).

**Table 2:** Univariate and multivariate Cox regression analyses of prognostic factors in patients with PD in training set.

**Variable**	**Univariate analysis**		**Multivariate analysis**	
	**HR (95%CI)**	**P-value**	**HR (95%CI)**	**P-value**
Age	1.056(1.015-1.099)	0.007	1.049(1.003-1.098)	**0.038**
Sex				
Male	Reference		Reference	
Female	0.745(0.414-1.374)	0.357	0.679(0.373-1.302)	0.258
PD durations	1.085(1.036-1.136)	0.001	1.067(1.012-1.125)	**0.017**
HY				
≤3	Reference		Reference	
≥4	5.097 (2.682-9.686)	0.000	2.737(1.300-5.761)	**0.008**
MMSE	0.957(0.893-1.025)	0.208	1.062(0.978-1.153)	0.153
CRP				
<0.3(mg/L)	Reference		Reference	
0.3-0.8(mg/L)	1.053(0.443-2.504)	0.908	0.914(0.379-2.202)	0.840
>0.8(mg/L)	2.879(1.314-6.308)	0.008	1.840(0.806-4.198)	0.148
Albumin(mg/dL)	0.388(0.191-0.787)	0.009	0.537(0.252-1.145)	0.537
NSAIDs				
Non	Reference		Reference	
Current/ habitual	1.006(0.447-2.262)	0.989	0.874(0.375-2.034)	0.754

### Development of an individualized prediction model

**Figure 2:**
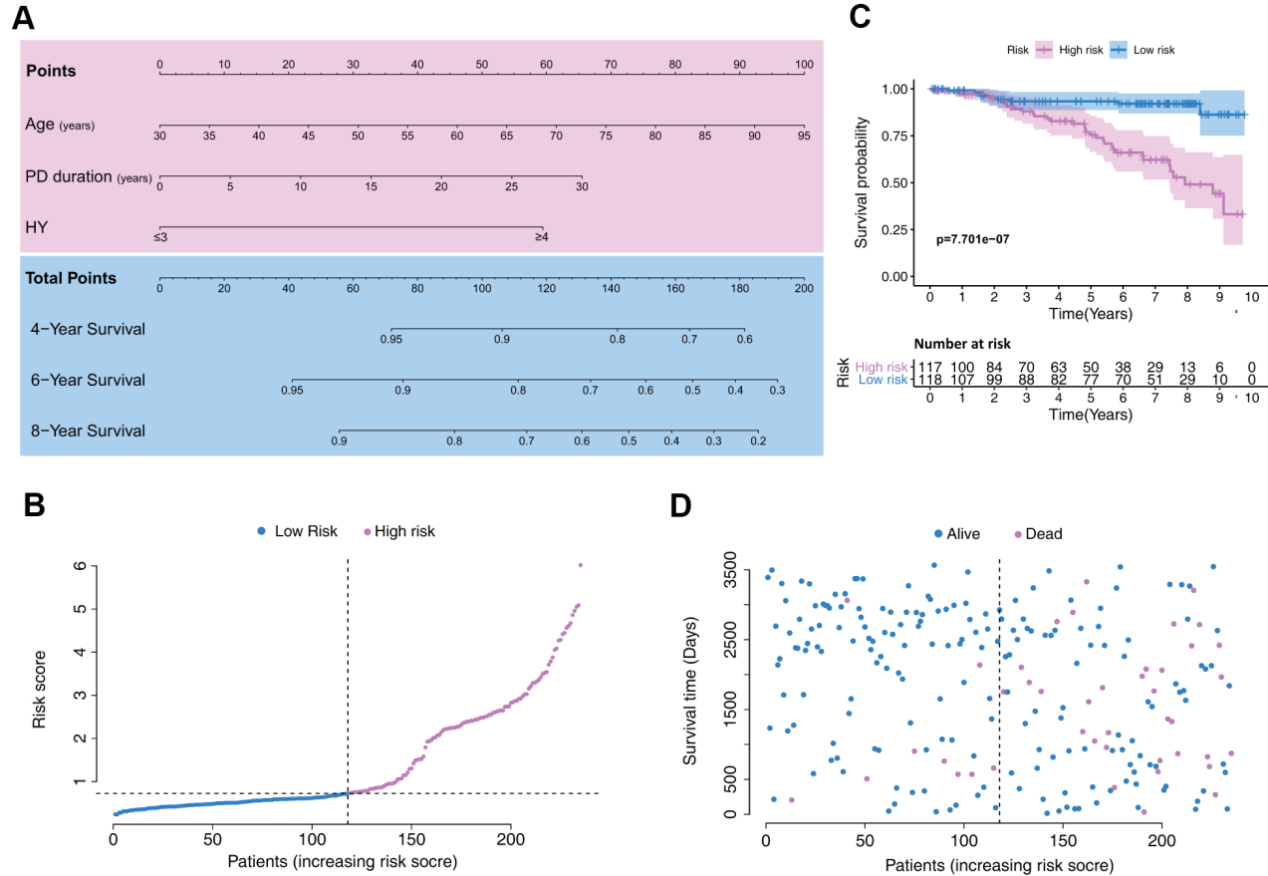
**Establishment of a model with clinical indices to predict Parkinson’s disease survival.** (**A**) A clinical feature model was used to develop a nomogram. (**B**) Distribution of the risk scores in the low- and high-risk groups. (**C**) Kaplan–Meier survival analysis between low- and high-risk groups. (**D**) Patient distribution in the low- and high-risk groups based on survival status.

Based on the Cox regression results, age, PD duration, and HY stage were incorporated into the nomogram (Figure 2A), which is an intuitive visualization model. According to the nomogram, age had the greatest influence on PD prognosis, followed by PD duration and HY stage. The total score is a sum of the individual scores of these three variables, and users finally obtain a specific probability of 4-, 6-, and 8-year survival.

Patient data included in the training set were clustered into high- (n = 117) and low-risk clusters (n = 118) according to the median risk score, following the risk score distribution shown in Figure 2B. The Kaplan–Meier survival curves exhibited significantly worse overall survival in the high-risk group (p=7.701e-7; Figure 2C). Training set patients’ survival time and status are shown in Figure 2D.

### Establishment of a web-based calculator

**Figure 3:**
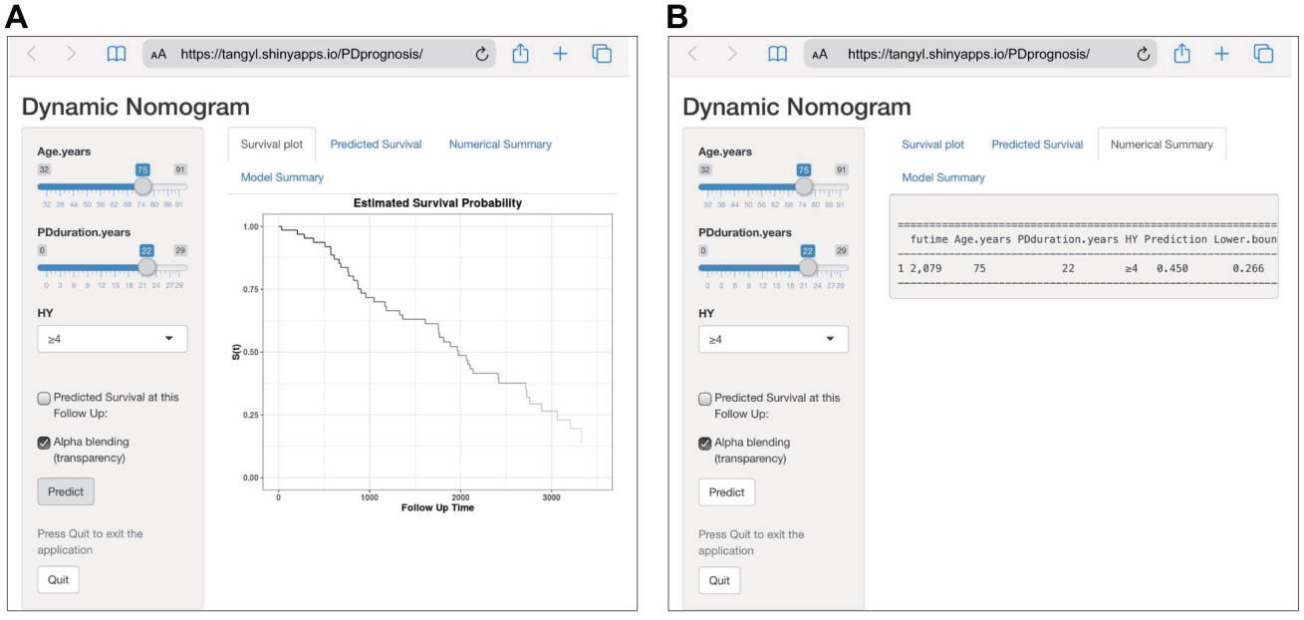
**Construction of a web-based calculator for predicting Parkinson’s disease survival based on the model (https://tangyl.shinyapps.io/PDprognosis/).** (**A**) Web survival rate calculator. (**B**) 95% confidence interval of the web survival rate calculator.

To facilitate the clinical application of our findings, we established a web-based calculator (https://tangyl.shinyapps.io/PDprognosis/) to predict the overall survival of patients with PD according to the nomogram (Figure 3). For example, for patients aged 75 years with 22 years of PD duration and an HY stage 4-5, the 2079-day survival rate was approximately 45.0% (95% CI 22.6–76.0%).

### Model performance in the training set

**Figure 4:**
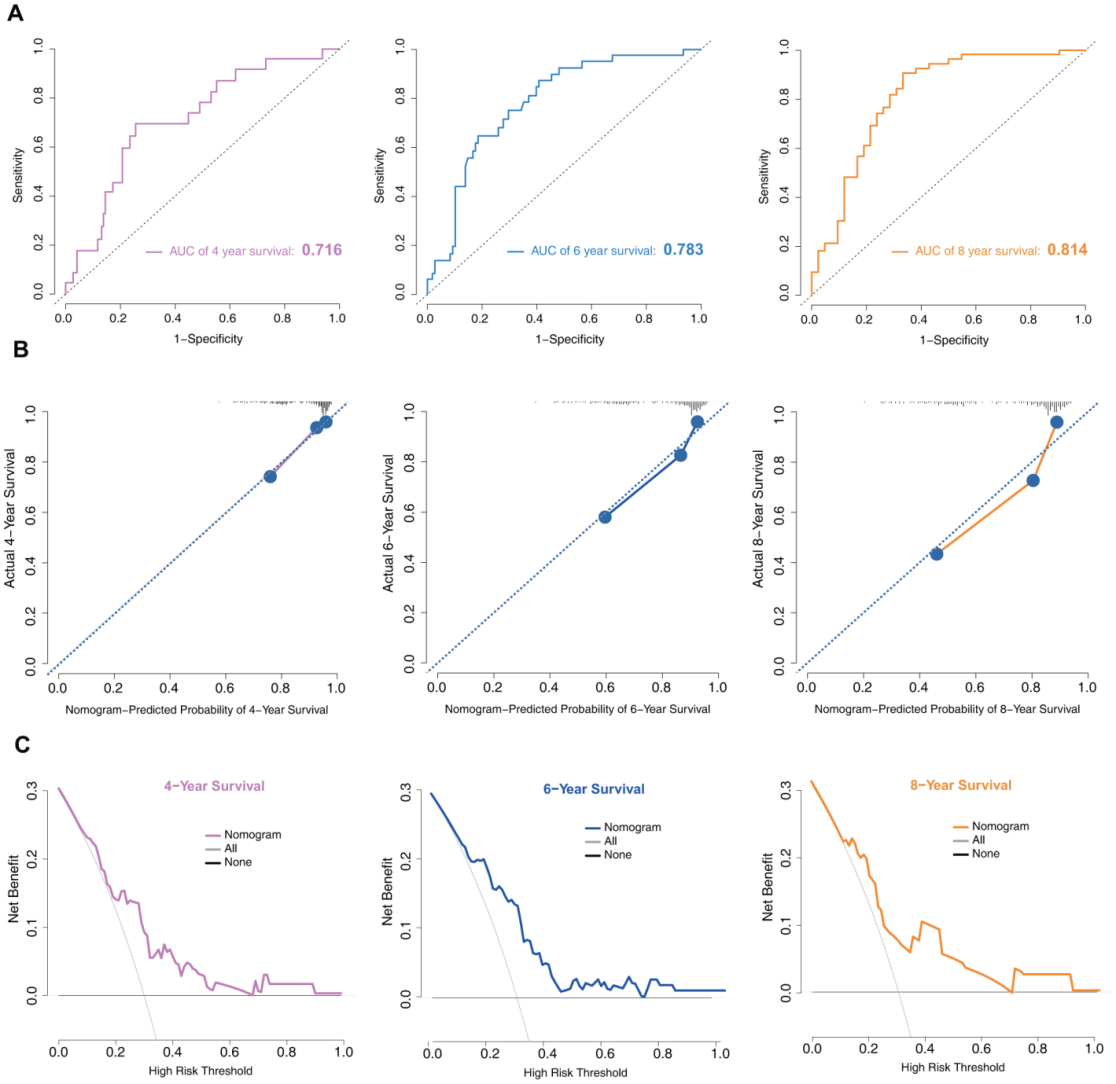
**Model discrimination and performance in the training set.** (**A**) Receiver operating characteristic curves for nomogram-based prognostic prediction. (**B**) Calibration plot examining estimation accuracy. (**C**) DCA assessing clinical utility.

In the training set, the discrimination power of the model was assessed by concordance index (C-index) values and receiver operating characteristic (ROC) curves. The C-index of the model was 0.75. The area under the curve (AUC) values generated to predict 4-, 6-, and 8-year survival were 0.716, 0.783, and 0.814, respectively (Figure 4A), implying that the nomogram was efficient in predicting prognosis. Calibration plots based on the training set showed good consistency between nomogram prediction and actual observation (Figure 4B).

Decision curve analysis (DCA) is a suitable method for evaluating alternative prognostic strategies that have advantages over other commonly used measures [15, 16]. Therefore, we applied DCA to evaluate the clinical usefulness of the prognostic nomogram. As shown in Figure 4C, for a >20% probability threshold, patients with PD would gain more benefit from this prognostic nomogram than the hypothetical treat-all or treat-none scenarios.

### Model performance in the validation set

**Figure 5:**
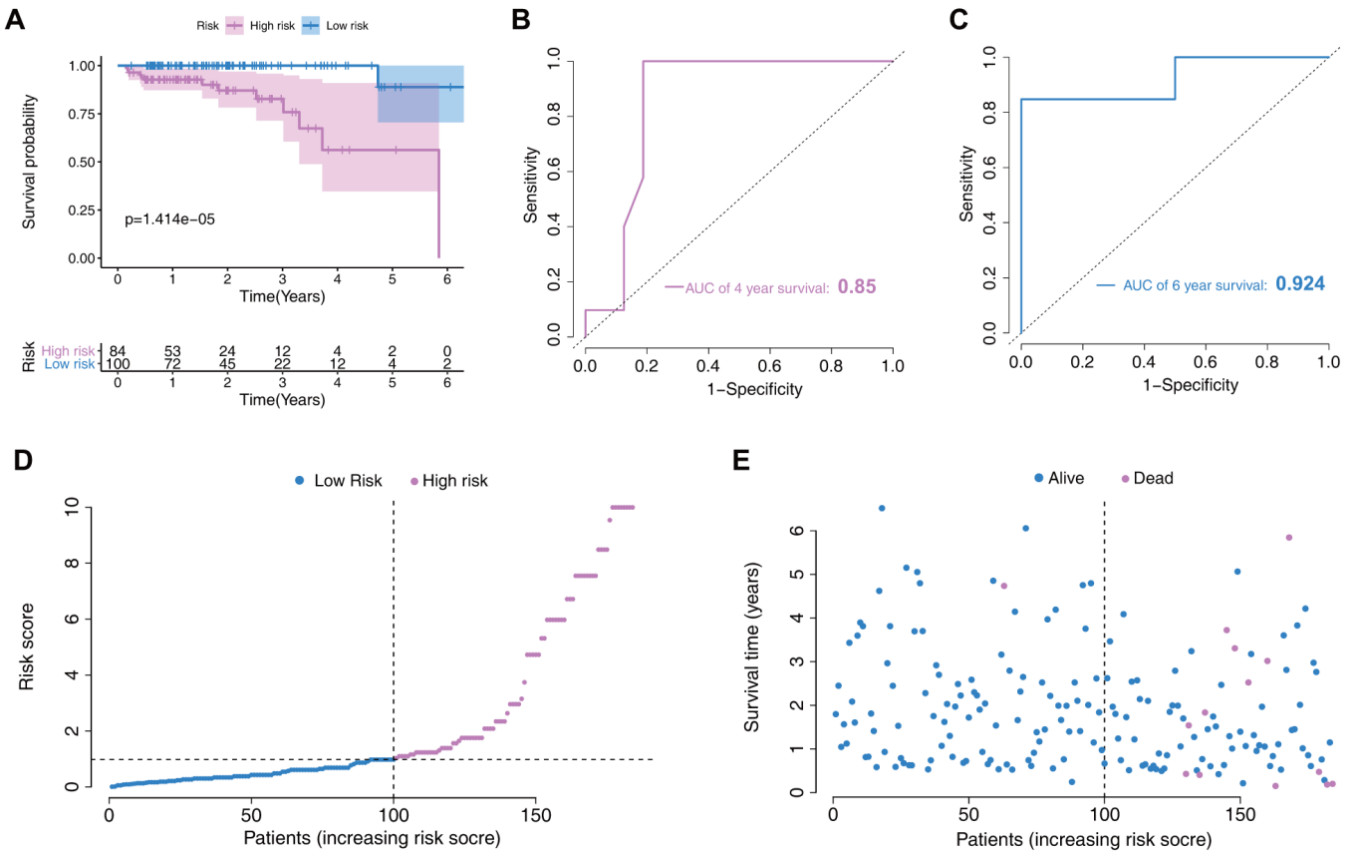
**Nomogram verification in the validation set.** (**A**) Kaplan–Meier survival analysis between the low- and high-risk groups. (**B**) Receiver operating characteristic (ROC) curves for 4-year prediction. (**C**) ROC curves for 6-year prediction. (**D**) Distribution of the risk scores in low- and high-risk groups. (**E**) Patient distribution in the low- and high-risk groups based on survival status.

In the validation set, risk-scores were calculated by the same formula used for calculating the patient risk-scores in the training set. Then, patients were divided into two low- and high- risk groups according to the model’s median score. As the longest follow-up time was 6.5 years in the validation set, we mainly assessed the prognostic value for 4- and 6-year survival probability. Survival analysis indicated that low-risk patients had significantly better prognosis than high-risk patients (p=1.414e-05, Figure 5A). Moreover, this new model showed good discriminative power in prognosis predictions, as reflected by an AUC for 4- and 6-year survival of 0.85 and 0.924, respectively (Figure 5B, 5C). The risk score distribution and the survival status of individuals in the low- and high-risk groups are shown in Figure 5D, 5E.

**Figure 6:**
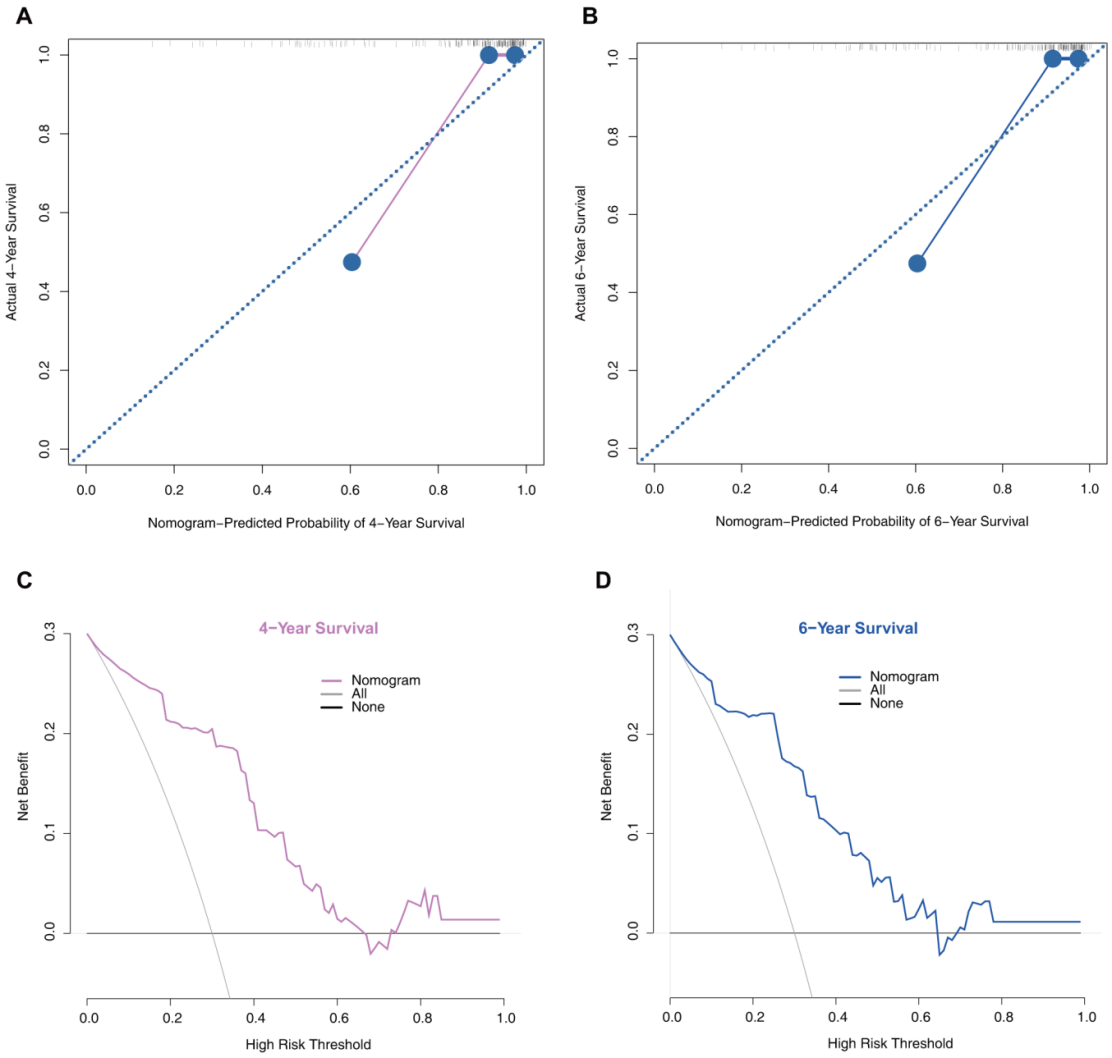
**Model performance in the validation set.** (**A**) 4-year calibration plot examining the estimation accuracy. (**B**) 6-year calibration plot examining the estimation accuracy. (**C**) 4-year decision curve analyses assessing clinical utility. (**D**) 6-year decision curve analyses assessing clinical utility.

Moreover, we performed calibration plot analysis as shown in Figure 6A, 6B the results of which suggested that this model has good probability consistencies between prediction and observation in the validation set. Similarly, using this model to predict PD survival has more net benefits (Figure 6C, 6D).

## DISCUSSION

PD is one of the most common neurodegenerative diseases and has heterogeneous clinical outcomes; hence, more accurate predictive models are required to guide treatment. As we know, there are many models for predicting disease progression in patients with PD. However, the survival prediction models of PD are limited. To the best of our knowledge, this is the first study to develop a nomogram and web-based calculator for predicting the survival of patients with PD.

In the present study, we established a model based on several readily available variables. The multivariable model based on three features (age, PD duration, and HY stage) showed the best predictive power in both training and validation sets. In the training set, the AUC of 4-, 6-, and 8-year survival was 0.716, 0.783, and 0.814, respectively. In the validation set, the AUC of 4- and 6-year survival reached 0.85 and 0.924, respectively. Model performance was also evaluated via calibration curves and DCA in both datasets. Our results suggest that the model could be used as a cost-effective tool to predict PD prognosis and assist with clinical decision-making.

Currently, researchers are exploring novel biomarkers for diagnosing and predicting mortality in patients with PD. While many studies have been conducted, few of these markers have been applied for predicting survival in patients with PD. Therefore, we choose easily obtainable variables that are convenient to apply in clinical practice. In the present study, we identified three independent risk factors, including age, PD duration, and HY stage, for predicting PD survival time. It has been established that age is a contributing factor in the progressive decline of dopamine transporter binding in healthy aging subjects [17]. PD is one of the most prevalent age-related neurodegenerative disorders, affecting 2–3% of the global population ≥65 years of age [18]. With an increase in age, the occurrence of PD also increases [19]. Thus, it has been regarded as the greatest risk factor for PD [20], and older age at baseline is associated with more rapid disease progression. Moreover, disease duration is also associated with PD progression [21, 22]. Nevertheless, there is evidence that age of PD patients contributes to disease severity, independent of the duration of the disease [23]. The HY scale is a widely used clinical standard for assessing PD stage, which is based on the level of clinical disability [24]. While not necessarily intimating pathophysiologic correlation, this method of staging is practical and allows for reproducible assessments by independent examiners of the general functional level of the patient. Combining these three clinical parameters, we developed a simple and accurate model.

Previous studies investigated single prognostic factors for PD [12, 25]. However, multivariable predictive models are known to show increased accuracy over individual risk factors. Therefore, unifactorial models are not appropriate for multifactorial diseases such as PD. Recently, studies have reported two models composed of several traditional factors [26, 27]. Macleod et al. reported a PD mortality model by combining four variables (age, sex, severity of axial features, and Charlson comorbidity index) with a moderate discrimination power (AUC=0.75 in the training set) [27]. Velseboer et al’s study identified a 3-variable predictor model with higher patient age, higher UPDRSME axial score, and lower animal fluency score, all giving a higher probability of an unfavorable outcome, with an AUC value was 0.765 [26]. However, there were several flaws in these two studies. First, they used a small sample size with less than 400 participants. Second, their models could only predict the survival probability for a single time-point.

Our study has several advantages when compared with previous studies. A major strength of this study was that the nomogram was developed and validated in two complete cohorts of patients diagnosed with PD of sufficient sample size. This is important as the samples of previous studies may have been too small, which might lead to inaccurate outcome predictions. We tested the nomogram’s discrimination power by an ROC analysis, finding that the AUC of the 8-year survival prediction in the training set reached 0.814, while that of the 6-year survival prediction in the validation set reached 0.924. The prediction accuracies significantly increased when compared to the previous study [26, 27]. Further, all three variables (age, PD duration, and HY stage) in our nomogram are easily available. Therefore, medical personnel can quickly perform an assessment without laboratory tests or instrumental examinations. Moreover, to facilitate clinical application, we established a web-based calculator to provide a free prediction service. It is convenient to provide the individual mortality percentages at different time-points. In addition, it cuts the cost and time needed for assessments both for PD patients and medical staff.

There are certain limitations to this study. First, eight probable prediction variables were included in the analysis, while other features, such as dementia, psychotic, and other symptoms, which may function as independent risk factors for PD survival, failed to be considered in this model. Second, as racial differences may contribute to different PD outcomes [28], and participants in this study only included those of Asian ethnicity, the conclusions from this study and this predictor might not be generalizable. Third, the primary outcome measure was patient survival; other outcomes, including postural instability or dementia, should be assessed in future studies.

In conclusion, we identified three variables and developed a novel nomogram and a web-based calculator to predict survival in patients with PD. These results may help further improve clinical decision-making and individualized treatment for PD patients. Furthermore, since this model could distinguish patients with high PD risk, it could help to closely follow-up on those patients.

## MATERIALS AND METHODS

### Patients

Raw data were extracted from a non-profit repository named the Dryad Digital Repository (http://www.datadryad.org/). This cohort study was conducted at the Utano National Hospital Parkinson’s Disease Centre. The study design comprised two stages. The first stage (training set) retrospectively identified patients with PD from March 2004 to November 2007 [14]. The enrollment criteria included PD patients free of any infection. The second stage (validation set) enrolled PD patients from July 2005 to July 2015 [29]. These patients underwent brain magnetic resonance imaging to exclude other neurologic disorders. Moreover, patients were excluded if they were undergoing tube feeding, had undergone tracheostomy, or had other diseases that could cause dysphagia. All PD patients were diagnosed according to the United Kingdom Parkinson’s disease Brain Bank Clinical Diagnostic Criteria. The study was approved by the Bioethics Committee of Utano National Hospital (No.28-15). Detailed information on the original studies is shown in Supplementary Table 1.

### Data collection

We performed a secondary analysis based on data from the above two stages. Eight probable prediction variables were selected, including age, sex, PD duration, HY stages, Mini-Mental State Examination (MMSE) scores, serum albumin, CRP, and nonsteroidal anti-inflammatory drug (NSAIDs) history. Moreover, survival time and survival status of each patient was extracted.

### Statistical analysis

To obtain a subset of predictors, univariate and multivariate Cox proportional hazards analyses were performed to select the optimal predictors from the risk factors in the training set. Variables with a p-value <0.05 in multivariable Cox regression were to act as independent predictors [30]. By using the selected variables based on the Cox regression results, a nomogram was developed using the “rms”, “survival”, and “foreign” R packages. In addition, the “DynNom” and “survival” R packages were used to construct a web-based calculator for predicting PD survival. The C-index and ROC curve were used to evaluate the discrimination ability of the model. Nomogram performance was assessed by a calibration plot and DCA, both in the training and validation cohorts.

Normal distribution data are expressed as means ± standard deviation, and non-normal data are expressed as medians (interquartile range). Differences between the training and validation sets were analyzed using chi-square tests for categorical variables and t-tests for continuous variables. The Kaplan–Meier method and the log-rank test were used to estimate survival. All analyses were performed with the software R (Version 3.6.2; http://www.Rproject.org). A value of p-value <0.05 was considered statistically significant.

### Data sharing

Data are available from the Dryad Digital Repository (http://www.datadryad.org/).

## Supplementary Material

Supplementary Table 1
