# Supplementary material for: A simple-to-use web-based calculator for survival prediction in Parkinson’s disease
Source: Aging (Albany NY). 2021 Feb 1;13(4):5238–49. doi: 10.18632/aging.202443 (PMC7950310; doi:10.18632/aging.202443)
Supplement: Supplementary Table 1 [file aging-13-202443-s001.pdf]

SUPPLEMENTARY TABLE

Supplementary Table 1. Details of the original studies.

	Stage I (training set)	Stage II (validation set)
N	313	184
Performed time	March 2004 to November 2007	July 2005 and July 2015
Enrollment criteria	PD were diagnosed according to the United Kingdom Parkinson’s disease Brain Bank Clinical Diagnostic Criteria	PD were diagnosed according to the United Kingdom Parkinson’s disease Brain Bank Clinical Diagnostic Criteria
Exclude criteria	Any infection	Other neurologic disorders;<br>Receiving tube feedings;<br>Had a tracheostomy;<br>Other diseases that could cause dysphagia;<br>Observed for fewer than six months after VFSS;
Data collection	CRP, age, sex, PD duration, HY, MMSE, albumin, NSAID use, follow- up time	Age, sex, PD duration, UPDRS-3 score, HY stage, BMI, MMSE, Serum albumin, Patients consuming processed diets, follow-up time
Study design	Kaplan-Meier curves for the cumulative incidence of death were obtained after dividing patients into two groups according to clinical features. The log-rank test was used to determine the associations between life prognosis and clinical factors.	The patients were divided into two groups: those who developed aspiration pneumonia within six months after VFSS (cases) and those who did not develop aspiration pneumonia (controls)
Outcome	Survival time	The development of aspiration pneumonia;<br>Survival time

VFSS: Video-fluoroscopic swallowing study.
